# Microencapsulation of cellular aggregates composed of differentiated insulin and glucagon-producing cells from human mesenchymal stem cells derived from adipose tissue

**DOI:** 10.1186/s13098-020-00573-9

**Published:** 2020-08-05

**Authors:** Claudia Jara, Felipe Oyarzun-Ampuero, Flavio Carrión, Esteban González-Echeverría, Claudio Cappelli, Pablo Caviedes

**Affiliations:** 1 Programa de Farmacología Molecular y Clínica, ICBM, Facultad de Medicina, Universidad de Chile, Independencia 1027., Casilla 7, Clasificador Nº 7, 8389100 Santiago, Chile; 2 Advanced Center of Chronic Diseases (ACCDiS), Universidad de Chile, Santiago, Chile; 3 Depto. de Ciencias y Tecnología Farmacéuticas, Facultad de Ciencias Químicas y Farmacéuticas, Universidad de Chile, Santiago, Chile; 4 Programa de Inmunología Traslacional, Facultad de Medicina, Clínica Alemana Universidad del Desarrollo, Santiago, Chile; 5 Laboratorio de Patología Molecular, Instituto de Bioquímica y Microbiología, Facultad de Ciencias, Universidad Austral de Chile, Valdivia, Chile; 6 Centro de Biotecnología y Bioingeniería (CeBiB), Departamento de Ingeniería Química, Biotecnología y Materiales, Facultad de Ciencias Físicas y Matemáticas, Universidad de Chile, Santiago, Chile

**Keywords:** Adipose-derived mesenchymal stem cells, Cellular aggregates, Cellular differentiation, Cell therapy, Diabetes, Microencapsulation

## Abstract

**Background:**

In type I diabetes mellitus (T1DM) pancreatic β cells are destroyed. Treatment entails exogenous insulin administration and strict diet control, yet optimal glycemic control is hardly attainable. Islet transplant could be an alternative in patients with poor glycemic control, but inefficient islet purification and autoimmune response of patients is still a challenge. For these reasons, it is necessary to explore new cellular sources and immunological isolation methods oriented to develop T1DM cell-based therapies.

**Aims:**

We postulate human adipose-derived stem cell (hASC) as an adequate source to generate pancreatic islet cells in vitro, and to produce islet-like structures. Furthermore, we propose microencapsulation of these aggregates as an immunological isolation strategy.

**Methods:**

hASC obtained from lipoaspirated fat tissue from human donors were differentiated in vitro to insulin (Ins) and glucagon (Gcg) producing cells. Then, insulin producing cells (IPC) and glucagon producing cells (GPC) were cocultured in low adhesion conditions to form cellular aggregates, and later encapsulated in a sodium alginate polymer. Expression of pancreatic lineage markers and secretion of insulin or glucagon in vitro were analyzed.

**Results:**

The results show that multipotent hASC efficiently differentiate to IPC and GPC, and express pancreatic markers, including insulin or glucagon hormones which they secrete upon stimulation (fivefold for insulin in IPC, and fourfold for glucagon, compared to undifferentiated cells). In turn, calculation of the Feret diameter and area of cellular aggregates revealed mean diameters of ~ 80 µm, and 65% of the aggregates reached 4000 µm^2^ at 72 h of formation. IPC/GPC aggregates were then microencapsulated in sodium-alginate polymer microgels, which were found to be more stable when stabilized with Ba^2+^, yielding average diameters of ~ 300 µm. Interestingly, Ba^2+^-microencapsulated aggregates respond to high external glucose with insulin secretion.

**Conclusions:**

The IPC/GPC differentiation process from hASC, followed by the generation of cellular aggregates that are later microencapsulated, could represent a possible treatment for T1DM.

## Background

Type I diabetes mellitus (T1DM) is an autoimmune and degenerative disease characterized by an autoimmune reaction against pancreatic β cells [1]. Glycemic control by conventional parenteral insulin (Ins) therapy accompanied by strict diet and controlled physical exercise is often inefficient, as the loss of β cells entails the absence of paracrine regulation between α and β cells [2].

A promising therapy had been proposed by results from the Edmonton protocol, which utilized glucocorticoid-free immunosuppression combined with infusion of an adequate mass of freshly prepared post-mortem pancreatic islets. With this treatment, 44% of treated subjects became insulin independent after 1 year of transplantation [3]. However, at present, this therapy is restricted to patients with poor or no metabolic control [4, 5]. Indeed, the shortage of donors and technical limitations entailed in pancreatic islet isolation make the massive implementation of this therapy difficult [6]. Also, activation of the immune system against the transplanted islets results in the gradual death of the graft [6]. Further, immunosuppressants used, such tacrolimus and/or sirolimus, are diabetogenic, and reportedly have other undesirable side effects such as mouth ulcers, diarrhea and acne [7]. Recently, new immunosuppression protocols have been tested, which include different T and B-cell depleting agents [8]. In addition to the use of long-term glucocorticoid-free immunosuppressants, immunological isolation of the graft by encapsulation has been proposed as a solution [6]. On the other hand, encapsulation of pancreatic islets provides a physical barrier against immune system activation [9]. Thus, it is essential to find new cellular sources and immunological isolation methods to face these limitations.

As a cellular source, we propose the use of mesenchymal stem cells (MSC), a population of multipotent cells present in almost all adult tissues [10]. MSC cells derived from human adipose tissue, or “adipose-derived stem cells” (hASC) are easier to purify by using less invasive methods [11, 12]. Previous studies showed that hASC can be differentiated to insulin producing cells (IPC) [13, 14], and ESC can be differentiated to glucagon producing cells (GPC) [15]. Other groups have reported generation of islet-like cell aggregates (ICA) [16, 17] to reestablish not only β-cell function, but also part of the necessary paracrine regulation between α and β cells [18]. However, the activation of the immune system against grafts remains a major limitation for this approach.

On the other hand, cellular encapsulation is a new strategy to provide immune isolation to avoid host rejection of grafts. Among the current existing techniques, microencapsulation entails several benefits: it allows the encapsulation of single islets by generation of spherical gels of micrometric size and with optimal surface/volume ratio, as compared to macroencapsulation systems [19]. Microcapsules indeed facilitate a rapid exchange of oxygen, glucose, insulin and nutrients between the capsule and the environment [19]. One of the most used biomaterials for encapsulation is sodium alginate, a natural copolymer that changes its physical state, from liquid to gel, in presence of divalent cations (mainly Ba^2+^ or Ca^2+^) [19]. It has been reported that encapsulation of islets in Ba^2+^-alginate preserve islet function in vitro with similar regulated insulin secretion compared to non-encapsulated islets [19]. In addition, Safley et al. demonstrated in vivo biocompatibility of Ba^2+^-alginate microgels [20], and others in vivo studies have demonstrated that Ba^2+^-alginate microgels provide long term immunoprotection in animal models [21–23]. In previous studies, successful allotransplant of parathyroid tissue encapsulated in Ba^2+^-alginate was reported in a patient suffering of severe hypocalcemia due to an iatrogenic parathyroid resection. In this case, the grafted tissue showed functionality for at least 20 months with a mild requirement of oral calcium supplementation [24, 25]. Considering this same principle, we hypothesized that we could generate functional, microencapsulated IPC/GPC aggregates in vitro, from IPC and GPC differentiated from hASC.

## Materials and methods

### Isolation and cell culture of hASC

This study was approved by the Research Ethics Committee, Faculty of Medicine, University of Chile. Adipose tissue was obtained from five donors aged between 30 and 45 years undergoing abdominal liposuction cosmetic surgery. Informed consent was obtained from all participants. hASC were obtained from lipoaspirated fat tissue, the tissue was digested by type I collagenase 0.2% p/v (Worthington, NJ, USA. Catalog number LS004196) and the stromal vascular fraction (SVF) was cultured in DMEM:F12 1:1 (Gibco™, Dublin, Ireland. Catalog number 12500062) medium supplemented with 10% v/v fetal bovine serum (FBS), denominated supplemented medium, at 37 °C and 5.0% CO_2_.

### Differentiation to IPC from hASC in vitro

**Fig. 1:**
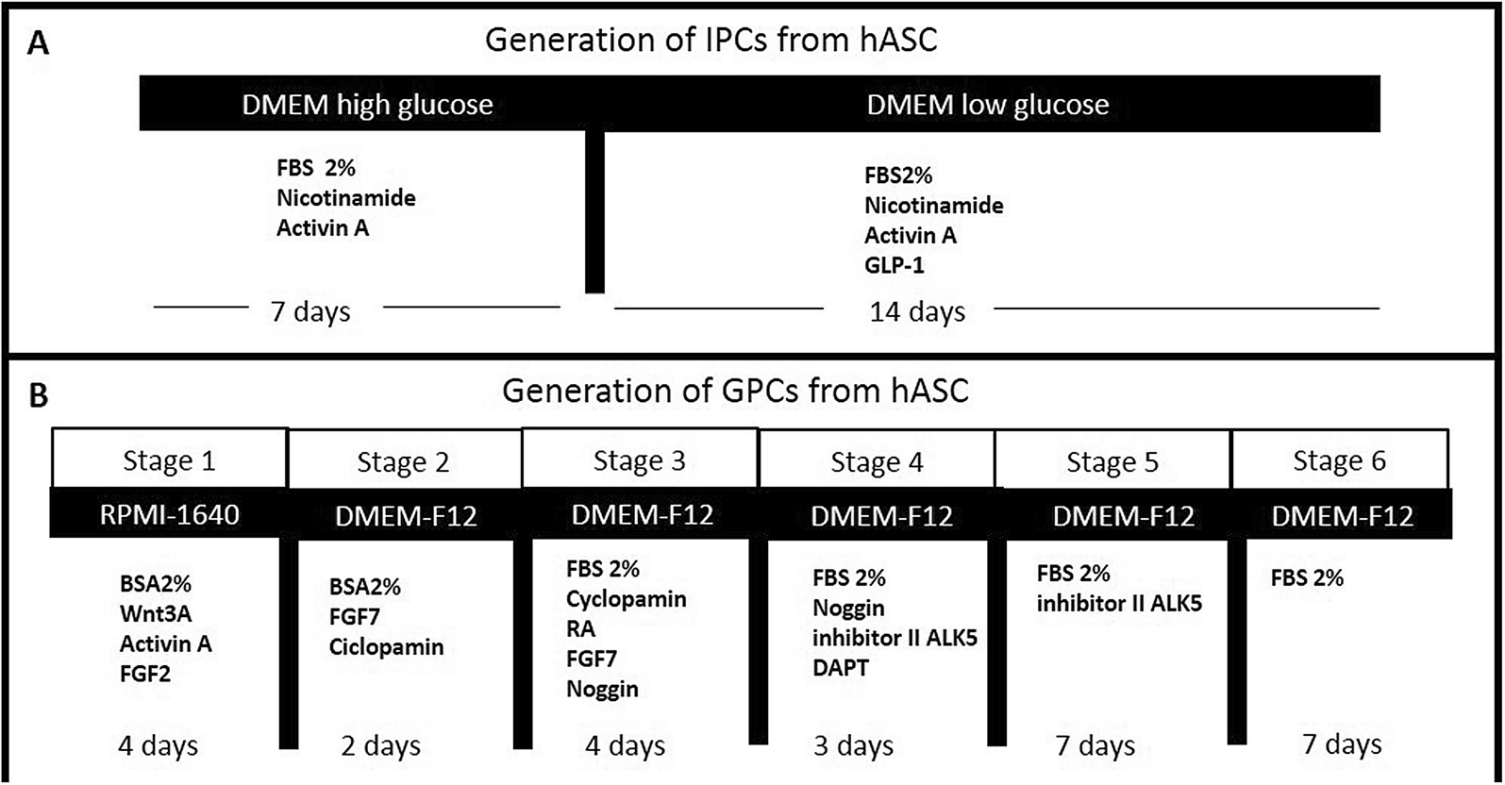
Schematic representation of differentiation protocols from hASC to IPC and GPC. **a** IPC differentiation protocol in two stages during 21 days. **b** GPC differentiation protocol in six stages during 27 days. FBS: fetal bovine serum, GLP-1: glucagon like-peptide 1, BSA: Bovine serum albumin, FGF2: fibroblastic growth factor 2, RA: retinoic acid, FGF7: fibroblastic growth factor 7, ALK 5: TGFβ type I receptor kinase, DAPT: Gamma-Secretase Inhibitor IX (*N*-[*N*-(3,5-Difluorophenacetyl-l-alanyl)]-S-phenylglycine t-butyl ester)

hASC were differentiated in vitro to IPC using a two-stage protocol [13, 14], with discrete modifications. Cells were stimulated for 7 days with DMEM high glucose (25 mM) (Gibco™, Dublin, Ireland. Catalog number 12100046); followed by 14 days with DMEM low glucose (5 mM) (Gibco™, Dublin, Ireland. Catalog number 31600034). Both media were supplemented with 2 nM activin A (Prospecbio, Rehobot, Israel. Catalog number CYT-145), 10 mM nicotinamide (Sigma-Aldrich, Merck KGaA, Darmstadt, Germany. Catalog number N0636) and 10 nM glucagon like peptide (GLP-1) (Prospecbio, Catalog number HOR-284). However, we used 2% v/v FBS instead of 10% v/v as a supplement to differentiation medium during all differentiation stages, Fig. 1a.

### Differentiation to GPC from hASC in vitro

GPC were obtained by the protocol reported for Rezania et al. [15] with modifications. The protocol consists of 6 stages: stage 1, incubated for 4 days with RPMI 1640 (Gibco™. Catalog number 31800), bovine serum albumin (BSA) (Sigma-Aldrich, catalog number A9418) 2%, activin A 100 ng/mL (Prospecbio), Wnt3a 20 ng/mL (Sigma-Aldrich. Catalog number H17001), FGF2 8 ng/mL (Prospecbio. Catalog number CYT-085). Stage 2, incubated for 2 days with DMEM-F12 medium with BSA 2%, FGF7 50 ng/mL (Prospecbio. Catalog number CYT-303), Cyclopamine-KAAD 0.25 µM (SCBT, Dallas, Tx, USA. Catalog number sc-200929). Stage 3, incubation for 4 days with DMEM-F12 medium, Cyclopamin-KAAD 0.25 µM, retinoic acid (RA) 2 µM (Sigma-Aldrich. Catalog number R2625), FGF7 50 ng/mL (Prospecbio), Noggin 100 ng/mL (Prospecbio. Catalog number CYT-475). Stage 4, incubation for 3 days with DMEM:F12 medium, Inhibitor II ALK5 1 µM (SCBT. Catalog number sc-221234), Noggin 100 ng/mL, DAPT 1 µM (SCBT. Catalog number sc-201315). Stage 5, incubation for 7 days with DMEM/F12 medium, inhibitor II ALK5 1 µM. Stage 6, incubation for 7 days with DMEM F12 medium. However, we used 2% v/v FBS instead of 1% B27 as a supplement to differentiation medium during stages 3 to 6, Fig. 1b.

### Formation of cell aggregates

The formation of cells aggregates was induced applying a proprietary protocol that subjects cells to low adherence conditions and progressive clustering, as previously described [26]. Briefly, differentiated IPC and GPC cells were incubated in a 4:1 ratio respectively, using 10^5^ total cells, under conditions of non-adherence. This protocol combines (1) the use of bacteriological plates with low electrical charge, and (2) culturing in low Ca^2+^ and Mg^2+^ using a special formulation of Eagle Minimum Essential Medium, Joklik Modification (Sigma-Aldrich. Catalog number M0518) and (3) reduced concentration of FBS (2% v/v) for up to 7 days.

### Immunofluorescent staining

#### Immunofluorescence of adherent cells

Undifferentiated and differentiated cells were grown in glass coverslips and fixed for 10 min with 4% paraformaldehyde (PFA) (Sigma-Aldrich. Catalog number 158127) in phosphate buffer saline (PBS) at room temperature, washed, and then permeabilized with 0.3% triton X-100 (Sigma-Aldrich. Catalog number T8787) for 10 min and incubated with 3% BSA in PBS (to block non-specific binding) for 45 min. Cells were then incubated overnight at 4 °C with Abcam primary antibodies (Abcam, Cambridge, UK) (Ms anti-C44 Catalog number ab6124, Goat anti-Pdx1 Catalog number ab47383, Rb anti-Ngn3 Catalog number ab38548, GPig anti-Ins Catalog number ab7842, Mouse anti-Gcg Catalog number ab 10988, and Goat anti-Vim Catalog number V-4630 (Sigma-Aldrich). Cells were then washed with PBS and incubated with corresponding secondary antibodies for 90 min at RT (Dnk anti-mouse Catalog number ab96875, Dnk anti-goat Catalog number ab96933, Dnk anti-rb Catalog number ab96919, Dnk anti-GPig Catalog number ab150185, Goat anti-mouse Catalog number ab6787, Dnk anti-goat Catalog number ab96931) (Abcam). Cells were washed with PBS and then incubated with Hoechst (Millipore, Burlington, MA, USA) 1:500 for 5 min at RT. Covers were washed and mounted in slides with fluorescence mounting medium DAKO (Agilent, CA, USA. Catalog number S302380). The images were analyzed using confocal microscopy (LSM700, Zeiss) and the ImageJ public domain software.

#### Immunofluorescence of cell aggregates

Prior to immunofluorescence, cell aggregates formed after 72 h were fixed in 4% PFA for 1 h and added to 1% liquid low melting agarose (Sigma-Aldrich, Catalog number A9414) in PBS (50 °C). To obtain cell blocks, the agarose-cell mix was centrifuged at 1500 rpm for 5 min at RT. Cryostat sections of 6 µm were submitted to conventional IFI and visualized by confocal microscopy (LSM 700, Carl Zeiss).

### ELISA assay

To test if the hormonal release of IPC, GPC cells and aggregates was glucose-dependent, two glucose concentrations (2 mM and 25 mM) were assayed. After pre-incubation with Krebs–Ringer buffer (KRB) without glucose (120 mM NaCl, 5 mM KCl, 2,5 mM CaCl_2_, 1,1 mM MgCl_2_, 25 mM NaHCO_3_, 10 mM HEPES, 0.1% BSA) at 37 °C for 2 h, the cells were incubated with KRB containing 2 mM glucose at 37 °C for 4 h. To induce insulin release, the cells were incubated with KRB containing 25 mM glucose for another 4 h. Then, the respective conditioned media were collected and tested for the content of released insulin with human insulin (Mercodia, Uppsala, Sweden. Catalog number 10-1132-01) and human glucagon (Cusabio, Houston, TX, USA. Catalog number CSB-E09207h) ELISA kits.

### Microencapsulation of cell aggregates

Alginate microgels were automatically produced using the dripping technique. As a polymer, 1.5% (MW medium) sodium alginate (Sigma-Aldrich. Catalog number A2033) dissolved in 0.9% NaCl was used. The stabilizing solutions of barium and calcium chloride were  maintained at pH 7.0 and were composed of (in mM): BaCl_2_ (20), NaCl (115), and histidine (50), or CaCl_2_ (40), NaCl (85), HEPES (10). First, 1 mL of sodium alginate solution (1.5% w/v) was mixed with 2 × 10^4^ cellular aggregates previously filtered through 40 μm and 120 μm sieves. This anionic suspension was pumped using an Encapsulator B-395Pro (Büchi, Flawil, Switzerland), equipped with a 150 µm nozzle over a stirring barium or calcium chloride solution for 30 s, with a dripping flow of 32 mL/min, 3000 Hz frequency and 1000 V voltage, as previously reported [27, 28]. After its formation, the microgels were washed with 0.9% NaCl and incubated in DMEM/F12 medium supplemented with 10% FBS in cell culture conditions (37 °C, 5.0% CO_2_). Additionally, rupture of the microgels was evaluated by dissolving the alginate with a solution composed of solution composed of 100 mM sodium citrate, 10 mM MOPS and 27 mM NaCl according to reports from Wang et al. [29].

### Statistical analysis

For the experiments of cell aggregate formation and encapsulation of BSA or SpA proteins, statistical analysis was performed using one-way ANOVA followed by Student’s *t* test as post hoc. Student t test was used in Ins or Gcg release tests. Differences were deemed statistically significant for p-values of less than 0.05.

## Results

### Characterization of hASC

The SVF obtained from each patient was used as a source of MSC, denominated hASC, and characterized following the mesenchymal stem cell minimal criteria described by Dominici et al. [30]. To define these cells as a population of hASC, we validated the specific pattern of surface markers associated to these cells by flow cytometry (Additional file 1: Table S1). The results of hASC characterization obtained from 5 donors confirmed that these cells were positive for expression of CD90, CD73, CD105, CD44, and CD29, while lacking expression of CD45, CD34, CD19 and HLA-DR surface markers. Moreover, we tested the differentiation potential of these cells to adipogenic, osteogenic and chondrogenic phenotype, and confirmed that hASC were able to differentiate to these three lineages compared to undifferentiated hASC, as evidenced by the detection of known characteristic features of differentiation such as the presence of lipidic intracellular vacuoles in adipogenic cells, calcium deposition determined by Von Kossa staining in osteogenic cells; and finally, sulfated mucines detected by alcian blue staining in chondrogenic cells (Additional file 2: Figure S1).

### Generation and characterization of IPC and GPC

**Fig. 2:**
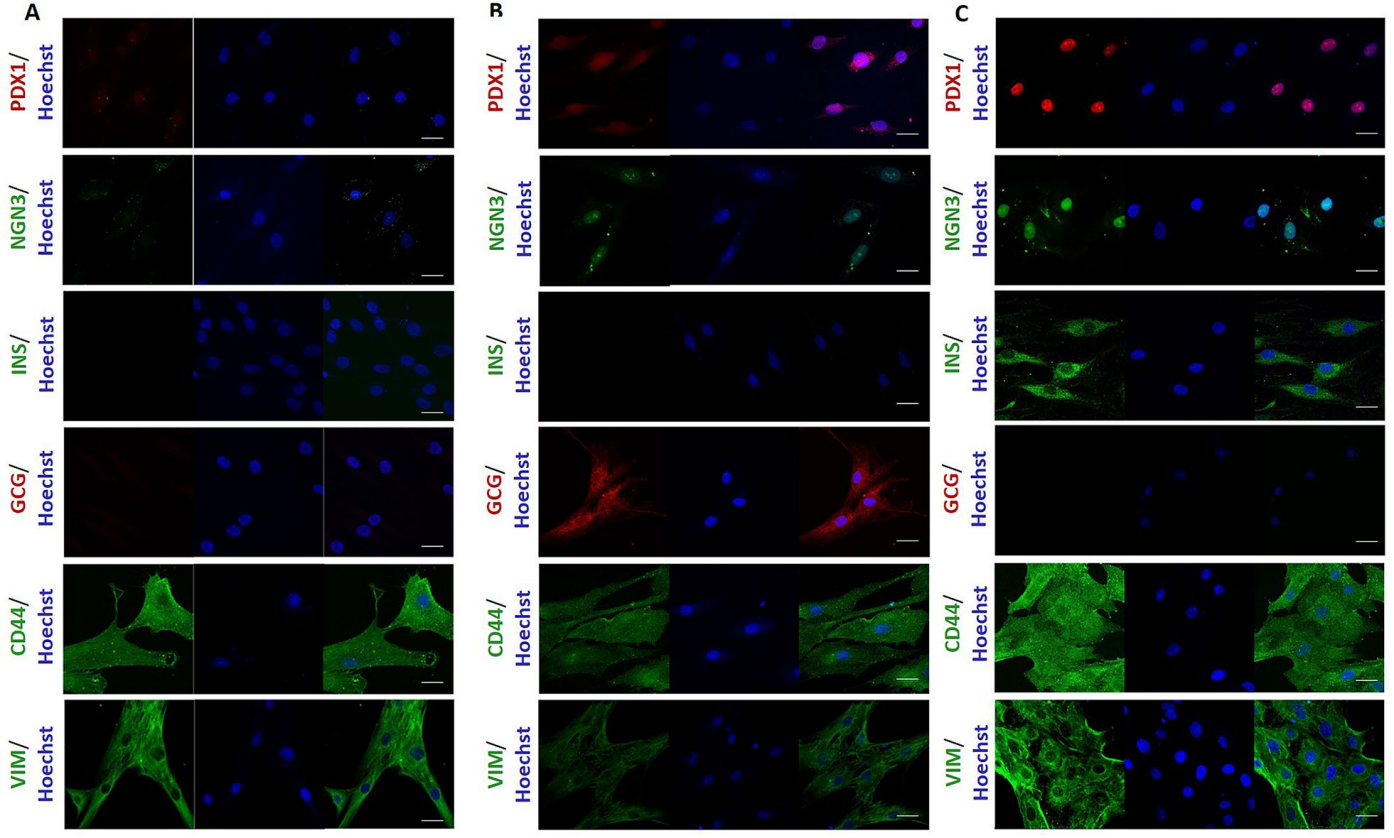
Expression of differentiation markers in hASC, IPC and GPC by indirect immunofluorescence. The images shown are representative of n = 3 experiments, visualized by confocal microscopy. Nuclear staining was performed with Hoechst 1: 500 (blue). Markers analyzed were Pdx1 (red), Ngn3 (green), Insulin (green), Glucagon (red), CD44 (green) and Vimentin (green). **a** Markers analyzed in hASC. **b** Markers analyzed in IPC. **c** Markers analyzed in GPC. Scale bar = 20 μm. *Pdx1* pancreatic and duodenal homeobox 1, *Ngn3* neurogenin 3, *Ins* insulin, *Gcg* glucagon, *Vim* vimentin

**Fig. 3:**
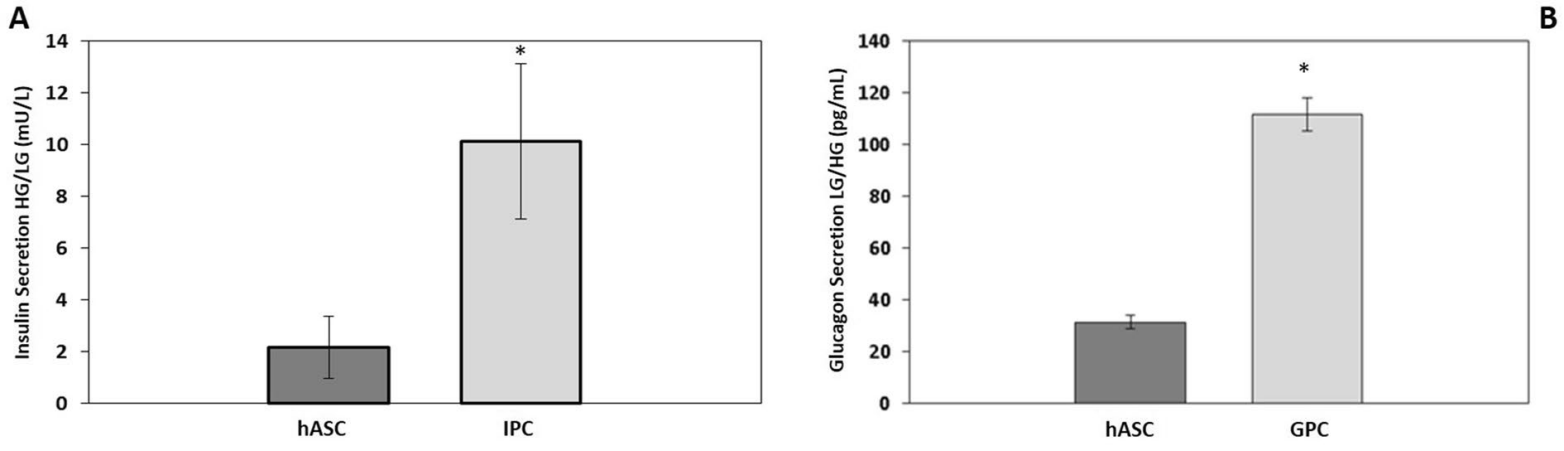
Hormonal secretion in response to low or high external glucose concentration in vitro. Glucose challenge was performed by incubating the cells with low-glucose buffer (2 mM) or high-glucose buffer (25 mM) for 4 h. **a** Insulin secretion in response to high glucose in vitro. **b** Glucagon secretion in response to low glucose in vitro. n = 3. *Significant p < 0.05 (Student’s t test)

After application of the in vitro differentiation protocol, we performed immunofluorescence analysis in IPC and GPC, which showed presence and nuclear localization of β cell specific transcription factors as Pdx1 and Ngn3, Fig. 2. On the other hand, hASC, expressed these markers poorly, with diffuse distribution (Fig. 2a). Moreover, IPC expressed insulin, which was not evident in hASC (Fig. 2b). Further, insulin secretion by IPC treated with external high glucose (25 mM) increased fivefold compared to undifferentiated cells (Fig. 3a). Conversely, GPC cells express glucagon and secrete the hormone in response to low glucose concentration in the medium fourfold greater than undifferentiated cells, Fig. 3b. These results suggest that our differentiation protocol of hASC to IPC and GPC, strongly reproduce the functionality of these cells.

### Cell aggregate formation

**Fig. 4:**
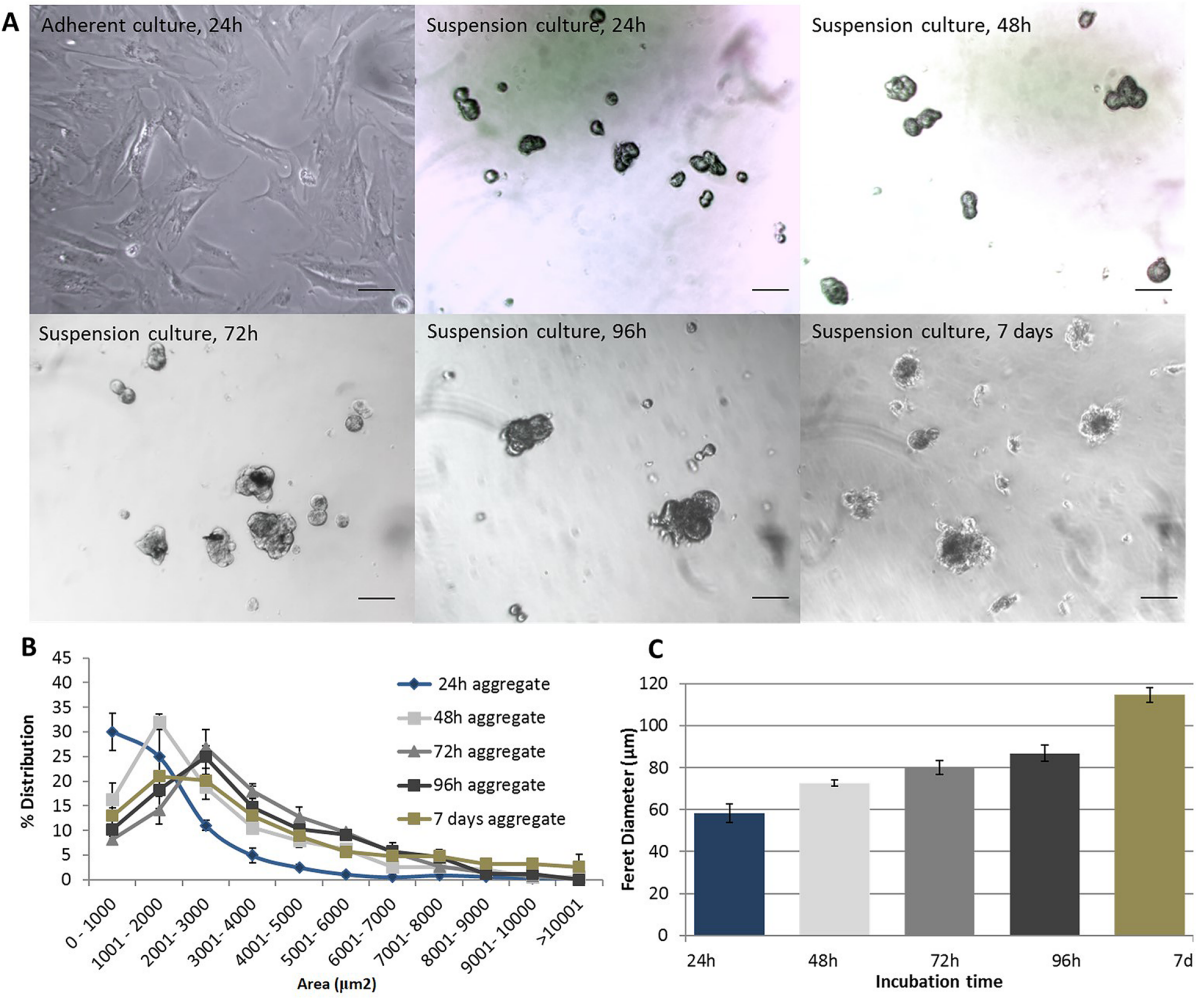
Time course of IPC/GPC aggregation formation. **a** Optical microscopy of IPC/GPC. **b** Area distribution of the cell aggregates. **c** Feret diameter of cells aggregates. All experiments were performed at 24, 48, 72, 96 h and day 7 of culture. ANOVA followed by Student’s t-test, *p < 0.05. n = 4. Scale bar = 100 μm

**Fig. 5:**
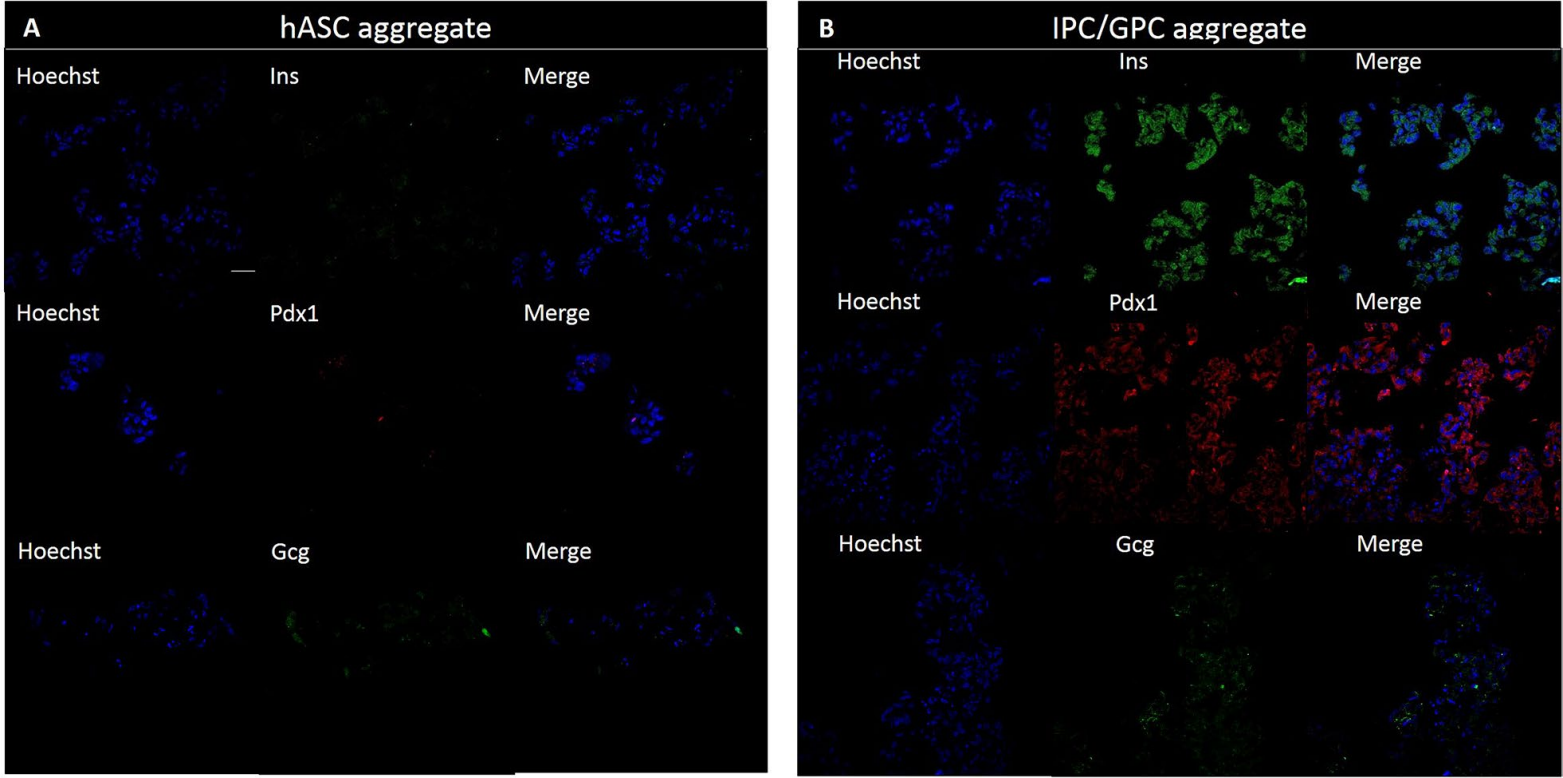
Expression of pancreatic markers in hASC and IPC/GPC aggregates at 72 h culture by indirect immunofluorescence. The images shown are representative of n = 3 experiments, visualized by confocal microscopy. The left panel corresponds to cell aggregates of hASC and the right panel to cell aggregates of IPC/GPC. **a** Insulin (green). **b** Pdx1 (red) and **c** Gcg (green). Hoechst staining 1:500 (blue) was performed to detect nuclei, n = 3. Scale bar = 40 μm

The results of IPC/GPC aggregate formation over time is shown in Fig. 4a. At 24 h of culture, aggregates are not yet evident. However, at 48 h almost all cells are aggregated. At 72 y 96 h, these formations were significantly larger. Finally, at day 7, the aggregates tend to lose their shape and cellular debris appears, suggesting cell death. The analysis by optical microscopy reveals that the area of the aggregates increased in time, Fig. 4b. At 24 h, we estimated that 55% of the aggregates have an area below 2000 µm^2^. At 48 h, most aggregates (65%) reached at least 2500 µm^2^. At 72 and 96 h, 65% of the aggregates reached 4000 µm^2^. By day 7, all aggregates comprised areas from 1000 to 4000 µm^2^, suggesting a slow decay of the aggregate structure at this time. Later, cell aggregates incubated at different times were filtered through 40 μm and 120 μm sieves to concentrate the aggregates within these sizes and evaluate the best time to obtain larger aggregates in a stable manner. When calculating the diameter, we found that at 72 h, most aggregates had similar diameters (~ 80 µm), Fig. 4c. Finally, confocal microscopy studies in cryostat sections of differentiated cell aggregates revealed the presence of Insulin and Pdx1, and very faint staining for glucagon (Fig. 5).

### Microcapsules of cell aggregates

**Fig. 6:**
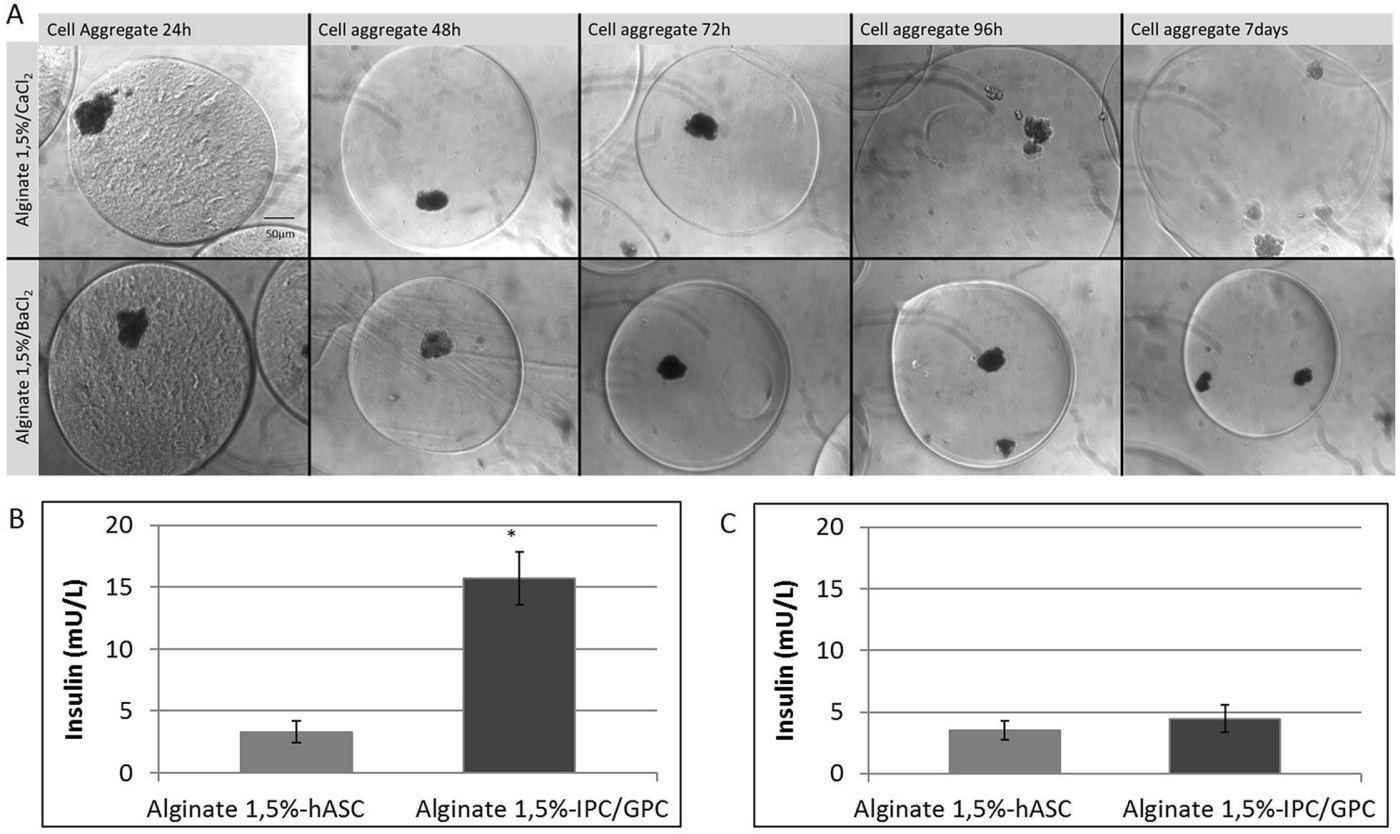
Characterization of IPC/GCP aggregates in microgels. Light microscopy was performed at different times for **a** Calcium (upper panel) or Barium (lower panel). (n = 5), **b** Insulin secretion from microgels at 1 day in HG conditions (n = 3), **c** Insulin secretion from microgels at 7 days in HG conditions (n = 3). Student’s t test, *p < 0.05. Scale bar = 50 μm

The aggregates were encapsulated in sodium alginate stabilized by Ca^2+^ o Ba^2+^ cations. Detection of alginate particle diameters by optical microscopy indicated that microgels generated were spherical, with an approximate diameter of ~ 310 µm (Fig. 6a and Table 1). To evaluate the permeability of microgels, SpA and BSA proteins (40 and 60 kDa, respectively) were previously encapsulated in 1.5% sodium alginate and stabilized by Ca^2+^ or Ba^2+^ cations, see Additional file 1. These microgels presented sizes similar (Additional file 1: Table S2) to those used to encapsulate cellular aggregates (Table 1). When then evaluated the encapsulation efficiency of our alginate microcapsules, which was estimated to be in the 54 and 58% range, that is, 54 to 58% of the total protein was encapsulated, whereas 46 to 42% of the protein remained in the stabilization medium without being incorporated into the microgel. No significant differences were found between encapsulated BSA or SpA proteins (Additional file 3: Figure S2).

**Table 1:** Diameter of microencapsulated cell aggregates in 1.5% sodium alginate

Sample	Diameter, time 0 (μm)		Diameter 7 days (μm)	
	CaCl_2_	BaCl_2_	CaCl_2_	BaCl_2_
Alginate 1.5%-hASC aggregate	310 ± 14	303 ± 23	459 ± 28 *	314 ± 15
Alginate 1.5%-IPC/GPC aggregate	302 ± 13	309 ± 12	468 ± 27 *	316 ± 9

Sizes of microgels were analysed by ImageJ in at least 10 photographs for each n, n = 3, *p < 0.05

Interestingly, when determining the release of SpA or BSA from the microgels to KRB, we observed a burst effect achieving up to of 13% of release in the first 5 min of incubation in both, Ca^2+^ or Ba^2+^ stabilized cations (Additional file 3: Figure S2). Then a steady state is reached at 15 and 20 min, achieving up to 16% of protein release in both, Ca^2+^ or Ba^2+^ microgels (Additional file 3: Figure S2), without finding significant differences between microgels formed with Ca^2+^ or Ba^2+^, nor between microgels containing SpA protein or BSA.

After IPC/GPC cellular aggregates encapsulation, we observed that cell aggregates microencapsulated in Ca^2+^ did not adequately stabilize alginate microgels, which began to swell and degrade after 96 h in culture conditions. Conversely, Ba^2+^ stabilized microgels were stable after 7 days (Fig. 6a). IPC/GPC Ba^2+^ stabilized microgels were capable of secreting insulin in response to elevated glucose concentrations (HG, 25 mM) at 1 day (Fig. 6b). However, after 7 days in vitro, this response decayed, and no significant differences were found thereon with respect to control (Fig. 6c).

## Discussion

Differentiation to IPC can be achieved from various cellular sources such as: embryonic stem cells (ESC) [31], induced pluripotent stem cells (iPSCs) [32] and MSC derived from adult tissue, as adipose (hASC) [13, 14], bone marrow (BM-MSC) [33], dental pulp (DPSCs) [34], and placenta [34]. Use of ESC and iPSCs in clinic is not routinely approved due to ethics and implementation considerations. Yet, hASC can be easily obtained from low invasive lipoaspiration procedures with high yield of cell purification. Hence, these cells constitute an important source of MSC [11, 12].

The hASC obtained in this work meet the requirements dictated by the International Society of Cell Therapy (ISCT) to classify a cellular population as a MSC [27, 35, 36]. Our cells were able to adhere to plastic with a fibroblastic phenotype, they expressed CD90, CD73, CD105, CD44, and CD29 while lacking expression of CD45, CD34, CD19 and HLA-DR, concordant to the surface marker expression described for hASC [11, 12]. Moreover, they were able to differentiate to adipogenic, chondrogenic and osteogenic phenotypes.

We then established a reliable method to induce differentiation of these cells into both IPC and GPC in vitro. Our results agree with those of other groups, in particular the differentiation process of hASC to IPC [13, 14]. On the other hand, differentiation to GPC has only been reported from ESC [15]. To the best of our knowledge, we are the first group to achieve an efficient and functional GPC differentiation from hASC using the protocol described by Rezania group, yet using SFB 2% instead of the B27 supplement. For GPC differentiation, we decided to use 2% FBS instead of 1% B27 as a supplement in the differentiation medium during stages 3 to 6 (Fig. 1b), a modification reached empirically, which provided more effective differentiation and greater survival, and it also matched the serum concentration used in our subsequent aggregation protocol. In line with these findings, we propose hASC as an adequate cellular source to generate IPC and GPC in cell-based therapy against T1DM.

Next, we co-cultured IPC and GPC in a 4:1 ratio by inducing cellular aggregation in low adhesion conditions, as previously described [26]. Using this setting, we observed that cell aggregates increased in size up to 72 h, reaching diameters of ~ 80 µm, similar to those reported for human islets (108 µm) [37]. Expression of α and β pancreatic cell markers in 72 h old cellular aggregates was also conserved, indicating a strong lineage commitment and low dedifferentiation rate of our cells.

Although human like islets should be able to partially restore the pancreatic endocrine function, the activation of the immune system is still a major limitation, together with the low number of donors for long term treatment [6, 8]. To overcome this issue, we encapsulated our IPC/GPC aggregates into sodium alginate microgels using an automatized encapsulator. The size of Ca^2+^ or Ba^2+^ stabilized sodium alginate microgels pools were very similar, with very low dispersion. In culture conditions, Ca^2+^ stabilized microgels increased in size and were less stable then Ba^2+^ stabilized microgels, which maintained their size and shape even after 7 days of culture. An explanation to this phenomenon lies in the chemical characteristics of the sodium alginate copolymer, which is composed by 1,4-b-d-mannuronic acid (M) and 1,4-a-l-guluronic acid (G) residues, and can be stabilized in general terms by divalent cations being Ba^2+^ the most efficient (Mg^2+^ ≪ Ca^2+^ < Sr^2+^ < Ba^2+^) [19].

Several studies have aimed to improve the output of encapsulated tissue grafts using sodium alginate. However, variables such as alginate purity and immunological response against residual contaminants left from the purification process are still a matter of discussion [19]. In our setting, both Ca^2+^ or Ba^2+^ stabilized alginate microgels were able to release both BSA and SpA proteins in the first 5 min, yet after 20 min the release rate diminished and remained stable. This information defined the alginate microgels pore size, which in essence permits the passage of nutrient such as glucose, as well as small peptides as Ins and Ggc. In agreement with these findings, we also confirmed that our microencapsulated cellular aggregates responded to external glucose by secreting Ins, thus confirming that the microgels allowed the passage of glucose into the microgel to be sensed by the cellular aggregates, which in turn responded by secreting insulin. Also, we observed that undifferentiated hASCs do exhibit a small secretion of insulin in response to external glucose stimulation, which is agreeable with previous reports that explain such phenomena as a result of possible basal, spontaneous differentiation, both in adherent as well as in suspension conditions [38]. It is therefore possible that a small percentage of cells kept in control conditions could have spontaneously differentiated into a pancreatic endocrine phenotype.

Despite the stability of the microgels, IPC/GPC aggregates ceased to secrete insulin after 7 days in culture. This may be attributable to loss of β cell function due to a dedifferentiation process, a desensitization of the IPC to glucose, or poor survival of the cellular aggregate in encapsulation conditions in the in vitro environment. Indeed, the described differentiation protocols attempted to reproduce the embryogenic process observed in organs and tissues in vitro. Yet the complexity of the embryogenic process in cell differentiation differs substantially from the much simpler in vitro milieu, a situation that could result in dedifferentiation, and in turn loss of phenotypic and functional characteristics. Nevertheless, an in vivo environment may overcome such limitations. We are currently planning experiments with in vivo implantation of our encapsulated clusters in NOD mice, an animal model of T1DM, which may shed light on this issue.

Our findings enforce the current knowledge on the potential of hASC as a source of multipotent cells in cellular therapy against T1DM. Moreover, we have developed a protocol to generate functional aggregates IPC/GPC like pancreatic islets, which can be encapsulated in alginate microgels to achieve immunoprotection. In such conditions, the encapsulated aggregates are stable and retain endocrine function in vitro for at least 7 days in culture.

## Conclusions

In the present work, we have achieved in vitro functional differentiation of human multipotent stem cells to IPCs and GPCs. Indeed, we have effectively attained IPCs and GPCs coaggregation to form islet-like clusters, and subsequently microencapsulation in alginate. Cell aggregates retain the capability of expressing endocrine pancreatic markers. Microencapsulation was standardized using a sterile, automated and highly reproducible procedure, thus optimizing protection from eventual immunological rejection after implants. Further, the microcapsules are capable of secreting insulin when subjected to external glucose stimulation.

Finally, the cell coaggregate formation conditions reported herein provide the basis for aggregation of any cell type, projecting its use in other pathologies where replacement of cell mass or function is required. Also, our findings may give rise to new studies, where the functionality of microgels and aggregates can be evaluated in vivo. In this way, the proposed therapy would, on the one hand, immunologically isolate cellular aggregates and, secondly, avoid immunosuppressive treatment.

## Supplementary information

**Additional file 1.** Materials and Methods. **Table S1.** Analysis of mesenchymal stem cell markers in hASC by flow cytometry. **Table S2.** Diameters of microencapsulated BSA and SpA proteins in 1.5% sodium alginate.

**Additional file 2: Figure S1.** hASC differentiation potential in vitro. Left, Oil red staining for adipogenic phenotype. Center, Von Kossa staining for osteogenic phenotype. Right, Alcian blue stain for condrogenic phenotype. For each staining, control undifferentiated cells (hASC) and differentiated cells using the stempro kit. n = 5. Scale bar = 10 μm.

**Additional file 3: Figure S2.** BSA or SpA release from microgels of sodium alginate 1.5% stabilized with Ca^2+^ or Ba^2+^ to KRB. n = 3, no significant differences were found, p > 0.05.

## Data Availability

All data generated or analyzed during this study are included in this published article and enclosed additional information files.
