# Correction to: Microencapsulation of cellular aggregates composed of differentiated insulin and glucagon-producing cells from human mesenchymal stem cells derived from adipose tissue

**DOI:** 10.1186/s13098-020-00581-9

**Published:** 2020-08-19

**Authors:** Claudia Jara, Felipe Oyarzun-Ampuero, Flavio Carrión, Esteban González-Echeverría, Claudio Cappelli, Pablo Caviedes

**Affiliations:** 1 Programa de Farmacología Molecular y Clínica, ICBM, Facultad de Medicina, Universidad de Chile, Independencia 1027., Casilla 7, Clasificador Nº 7, 8389100 Santiago, Chile; 2 Advanced Center of Chronic Diseases (ACCDiS), Universidad de Chile, Santiago, Chile; 3 Depto. de Ciencias y Tecnología Farmacéuticas, Facultad de Ciencias Químicas y Farmacéuticas, Universidad de Chile, Santiago, Chile; 4 Programa de Inmunología Traslacional, Facultad de Medicina, Clínica Alemana Universidad del Desarrollo, Santiago, Chile; 5 Laboratorio de Patología Molecular, Instituto de Bioquímica y Microbiología, Facultad de Ciencias, Universidad Austral de Chile, Valdivia, Chile; 6 Centro de Biotecnología y Bioingeniería (CeBiB), Departamento de Ingeniería Química, Biotecnología y Materiales, Facultad de Ciencias Físicas y Matemáticas, Universidad de Chile, Santiago, Chile

## Correction to: Diabetol Metab Syndr (2020) 12:66 10.1186/s13098-020-00573-9

Following publication of the original article [1], the authors identified an error in the caption of Fig. 2 and the in-text citation of Fig. 2. The error was that the descriptions for panel **b** and **c** were swapped.

**Fig. 2:**
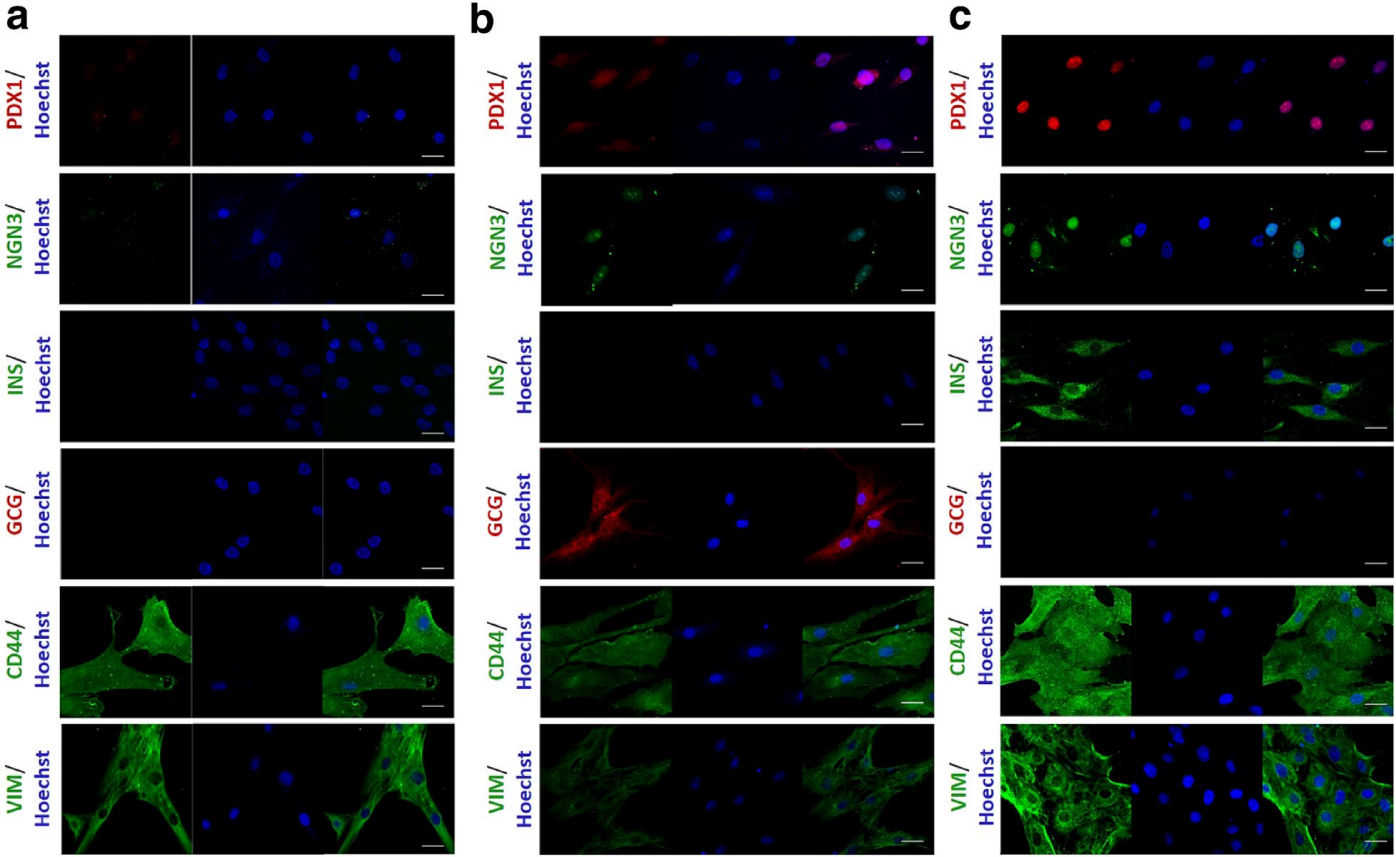
Expression of differentiation markers in hASC, IPC and GPC by indirect immunofluorescence. The images shown are representative of n = 3 experiments, visualized by confocal microscopy. Nuclear staining was performed with Hoechst 1: 500 (blue). Markers analyzed were Pdx1 (red), Ngn3 (green), Insulin (green), Glucagon (red), CD44 (green) and Vimentin (green). **a** Markers analyzed in hASC. **b** Markers analyzed in GPC. **c** Markers analyzed in IPC. Scale bar = 20 μm. *Pdx1* pancreatic and duodenal homeobox 1, *Ngn3* neurogenin 3, *Ins* insulin, *Gcg* glucagon, *Vim* vimentin

### Caption, page 5

- It currently reads “b Markers analyzed in IPC. c Markers analyzed in GPC.”
- It should read “b Markers analyzed in GPC. c Markers analyzed in IPC.”

### In-text citation, results section, page 5

- It currently reads: “Moreover, IPC expressed insulin, which was not evident in hASC (Fig. 2b)”.
- It should read “Moreover, IPC expressed insulin, which was not evident in hASC (Fig. 2c)”.

The figure with updated caption is published in this correction article.
