# Supplementary material for: Microencapsulation of cellular aggregates composed of differentiated insulin and glucagon-producing cells from human mesenchymal stem cells derived from adipose tissue
Source: Diabetol Metab Syndr. 2020 Aug 5;12:66. doi: 10.1186/s13098-020-00573-9 (PMC7409404; doi:10.1186/s13098-020-00573-9)
Supplement: Supplementary file 1 — Additional file 1. Materials and Methods. Table S1. Analysis of mesenchymal stem cell markers in hASC by flow cytometry. Table S2. Diameters of microencapsulated BSA and SpA proteins in 1.5% sodium alginate. [file 13098_2020_573_MOESM1_ESM.docx]

Additional Information

**2. Materials and Methods.**

- 1. **Characterization of hASC.** hASC in passages 2 to 5 were characterized for the expression markers of mesenchymal stem cells by flow cytometry. After trypsinization, detached cells were washed with PBS 1X and resuspended and then incubated with primary antibodies for 30 min at 4°C in darkness. After washing, cells were analyzed in a Coulter Epics flow cytometer using FCS Express 4 software. Antibodies for flow cytometric analysis were: IgG1k-FITC (Invitrogen, code MG101), IgG1k-PE (Invitrogen, code MG104), CD90-FITC (BD, code 555595), CD105-PE (BD, code 560839), CD45-FITC (Beckman C, code IM078211), CD34-PE (Beckman C, code IM1871U), CD44-FITC (BD, code 347943), CD73-PE (BD, code 550257), CD19-FITC (Invitrogen, code MHCD1901), CD29-PE (BD, code CD2904), IgG1k-AF488 (Invitrogen, code MG120), IgG2bk-PE (Invitrogen, code MG2B04), CD11b-AF488 (BD, code 557701), HLADR-PE (Invitrogen, code MHLDR04).
  2. **Assessment of multidifferentiation potential of hASC**. Adipogenic, chondrogenic and osteogenic differentiation potential was evaluated using commercials kits: Adipogenesis Differentiation Kit, Chondrogenesis Differentiation Kit and Osteogenesis Differentiation Kit (STEMPRO®, Gibco) following manufacturer’s instructions. After 21 days in culture, the multidifferentiation potential was confirmed by optic microscopy with chemical cell staining using Oil red for adipogenic phenotype, Alcian blue for chondrogenic phenotype and finally, Von Kossa staining for osteogenic differentiation.

**2.3 Microencapsulation of BSA and SpA proteins.** Alginate microgels were automatically produced using the dripping technique. As a polymer, 1,5% (MW medium) sodium alginate (Sigma- Aldrich) dissolved in 0,9% NaCl was used. The stabilizing solutions of calcium and barium chloride were maintained at pH 7.0 and were composed of (in mM): BaCl_2_ (20), NaCl (115), and histidine (50), or CaCl_2_ (40), NaCl (85), HEPES (10). First, the sodium alginate solution (1,5% w/v) was mixed with BSA (66 kDa) or Staphylococcal protein A (SpA) (40 kDa) 1 mg/mL. This anionic suspension was pumped using a Microencapsulator B-395Pro (Büchi, Switzerland) equipped with a 150 µm nozzle over a stirring barium or calcium chloride solution during 30 seconds, with a dripping flow of 32 mL/min, 3000 Hz frequency, and 1000 V voltage. After their formation, the microgels were washed with 0.9% NaCl and incubated under physiological conditions (37 °C, 5.0% CO_2_) in KRB (without BSA), to then evaluate the release of the protein from the microgel to the BKR at times 0, 5, 10, 15 and 20 minutes. In supernatant samples, proteins were quantified using the Bradford reagent, measured at 595nm. The encapsulation efficiency and percentage of protein released to the medium were analyzed according to a BSA calibration curve: 0-0.02-0.04-0.06-0.08-0.1- 0.2 mg / mL. Finally, alginate was dissolved as reported in [29] using a solution composed of 100 mM sodium citrate, 10 mM MOPS and 27 mM NaCl to corroborate the release results.

| Expression marker | % positive cells | | | | | | |
| --- | --- | --- | --- | --- | --- | --- | --- |
|  | hASC1 | hASC2 | hASC3 | hASC4 | hASC 8 | Average | SD |
| CD90 | 99.44 | 98.31 | 97.31 | 99.42 | 98.60 | 98.62 | 0.88 |
| CD105 | 99.31 | 99.40 | 99.42 | 99.5 | 94.50 | 98.16 | 2.44 |
| CD45 | 2.27 | 0.30 | 0.08 | 0.01 | 3.30 | 1.19 | 1.50 |
| CD34 | 11.67 | 6.60 | 1.20 | 0.15 | 3.50 | 4.62 | 4.65 |
| CD44 | 99.41 | 97.25 | 96.31 | 99.21 | 96.90 | 97.82 | 1.41 |
| CD73 | 99.29 | 99.18 | 99.88 | 99.44 | 99.20 | 99.40 | 0.29 |
| CD19 | 2.08 | 1.84 | 0.08 | 0.62 | 11.40 | 3.20 | 4.66 |
| CD29 | 99.80 | 99.92 | 99.96 | 99.43 | 95.90 | 99.00 | 1.75 |
| CD11b | 2.08 | 0.77 | 0.11 | 0.56 | 3.30 | 1.36 | 1.31 |
| HLADR | 0.04 | 5.19 | 0.13 | 0.26 | 2.80 | 1.68 | 2.27 |

**Table S1: Analysis of mesenchymal stem cell markers in hASC by flow cytometry**. The table presents cell marker studies from five different donors, carried out in subculture 2 to 5. 10,000 events were used per sample donor. The results are expressed as a percentage of positive cells. Labeled cells were read in a Beckman Coulter Epics cytometer and data were analyzed using the software FCS Express 4. Each tissue sample was studied using 10,000 events. SD: Standard deviation of the mean.

| Sample | Diameter time 0 (μm) | | Diameter 7 days (μm) | |
| --- | --- | --- | --- | --- |
|  | CaCl_2_ | BaCl_2_ | CaCl_2_ | BaCl_2_ |
| Alginate 1,5%-BSA protein | 294 ± 9 | 309 ± 13 | 329 ± 5 * | 296 ± 17 |
| Alginate 1,5%-SpA protein | 317 ± 14 | 285 ± 16 | 325 ± 4 | 286 ± 11 |

**Table S2: Diameters of microencapsulated BSA and SpA proteins in 1.5% sodium alginate**. Size of microgels analyzed by ImageJ of at least 10 photographs for each n. The figures represent means ± SEM, n=3. *p<0,05.
