# Supplementary figures and images for: Microencapsulation of cellular aggregates composed of differentiated insulin and glucagon-producing cells from human mesenchymal stem cells derived from adipose tissue
Source: Diabetol Metab Syndr. 2020 Aug 5;12:66. doi: 10.1186/s13098-020-00573-9 (PMC7409404; doi:10.1186/s13098-020-00573-9)

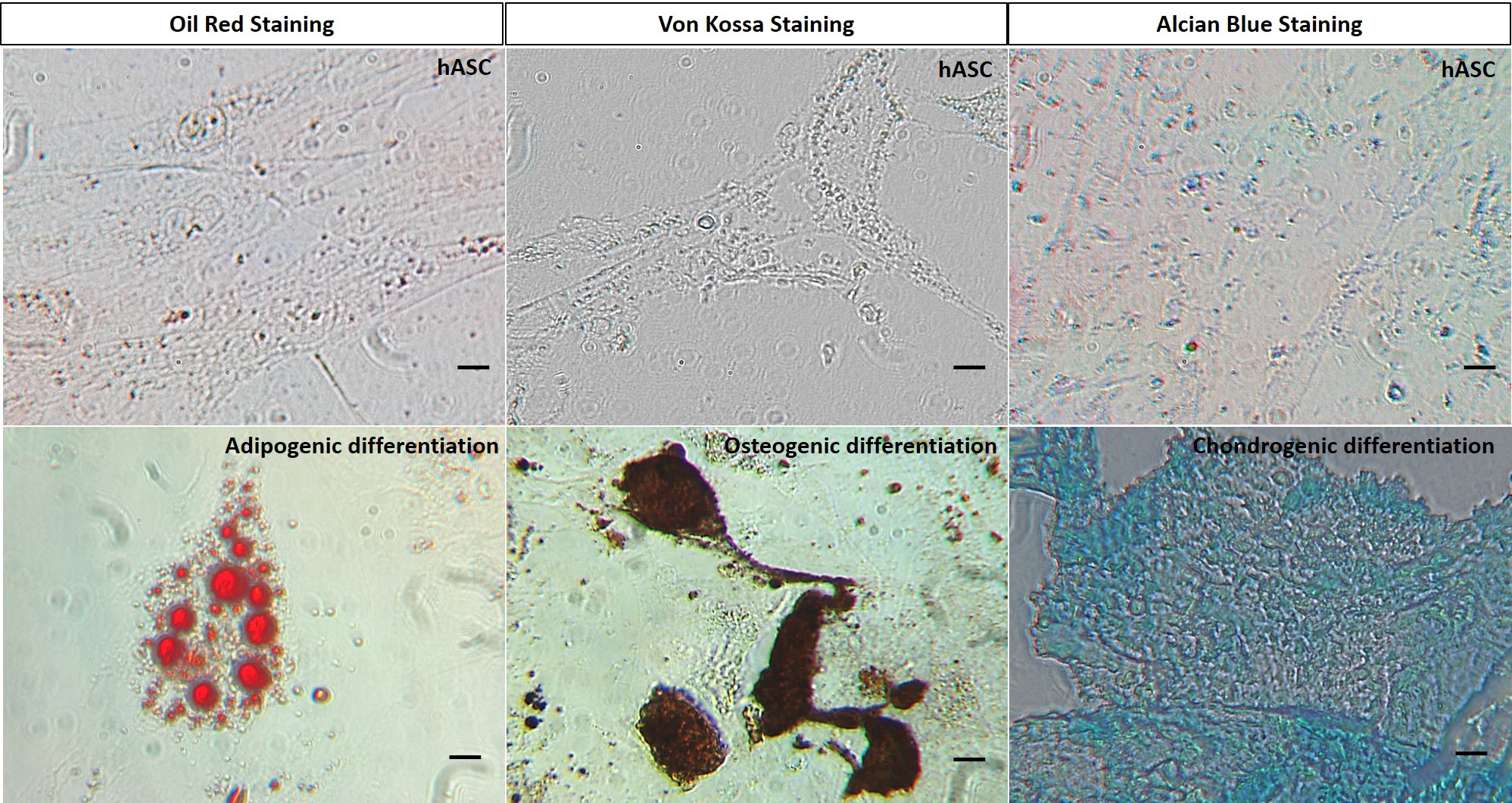

Supplement: Supplementary file 2 — Additional file 2: Figure S1. hASC differentiation potential in vitro. Left, Oil red staining for adipogenic phenotype. Center, Von Kossa staining for osteogenic phenotype. Right, Alcian blue stain for condrogenic phenotype. For each staining, control undifferentiated cells (hASC) and differentiated cells using the stempro kit. n = 5. Scale bar = 10 μm. [file 13098_2020_573_MOESM2_ESM.jpg]

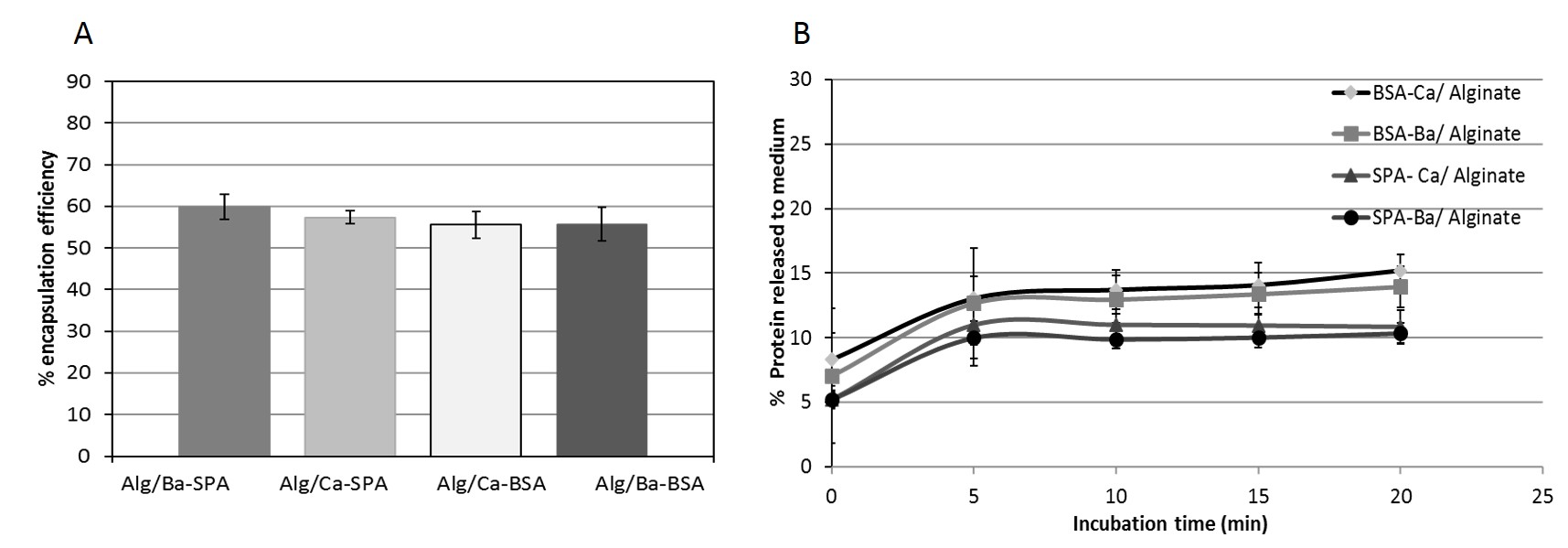

Supplement: Supplementary file 3 — Additional file 3: Figure S2. BSA or SpA release from microgels of sodium alginate 1.5% stabilized with Ca2+ or Ba2+ to KRB. n = 3, no significant differences were found, p > 0.05. [file 13098_2020_573_MOESM3_ESM.jpg]
